# The Facet Dependence of CO_2_ Electroreduction Selectivity on a Pd_3_Au Bimetallic Catalyst: A DFT Study

**DOI:** 10.3390/molecules28073169

**Published:** 2023-04-02

**Authors:** Ming Zheng, Xin Zhou, Yixin Wang, Gang Chen, Mingxia Li

**Affiliations:** 1 MIIT Key Laboratory of Critical Materials Technology for New Energy Conversion and Storage, School of Chemistry and Chemical Engineering, Harbin Institute of Technology, Harbin 150001, China; 2 School of Chemistry and Materials Science, Key Laboratory of Functional Inorganic Material Chemistry, Ministry of Education of the People’s Republic of China, Heilongjiang University, Harbin 150080, China

**Keywords:** electrocatalysis, CO_2_RR, density functional theory, facet dependence

## Abstract

The electrochemical carbon dioxide reduction reaction (CO_2_RR) has emerged as a promising approach to addressing global energy and environmental challenges. Alloys are of particular importance in these applications due to their unique chemical and physical properties. In this study, the possible mechanism of the C1 products from the electrochemical reduction of CO_2_ on four different surfaces of Pd_3_Au alloy bimetallic catalysts is predicted using the density functional theory. The differences in the number of d-band electrons and the charge distribution and morphology of the different surfaces result in differing catalytic activity and selectivity on the same surface. On different surfaces, Pd_3_Au alloy bimetallic catalysts have different potential limiting steps in CO_2_RR, resulting in differing selectivity. The Pd_3_Au (100) surface has a good selectivity for HER, indicating that the increase in the net charge on the surface of the alloy improves the selectivity for HER. The Pd_3_Au (211) surface, with a step structure, shows a good selectivity for methanol production from CO_2_RR. In addition, an electronic structure analysis shows that the selectivity of the reactions involved in the conversion of adsorbates is determined by the difference between the center of the d-band on the top of the catalyst, where the reactant and the product are located. The results of this study may provide some theoretical basis for designing and developing more efficient and selective CO_2_ reduction catalysts.

## 1. Introduction

The electrochemical reduction of carbon dioxide (CO_2_RR) has emerged as a promising approach to address the global energy and environmental challenges [1]. By using renewable electricity as its driving force, the electrochemical reduction of CO_2_ can convert a greenhouse gas into valuable chemicals and fuels, such as methane, ethylene, and formate [2]. In addition, it will also contribute to the storage of intermittent resources, such as solar, wind, and geothermal. Compared to traditional chemical processes, the electrochemical reduction of CO_2_ offers several advantages, including mild reaction conditions, a high selectivity, and renewable energy utilization. Therefore, it has attracted great attention in recent years and become a hot research topic in the fields of energy and environmental science. To apply this technology within industries, it is necessary to efficiently produce high-reaction-rate products. However, the development of efficient and selective electrocatalysts for CO_2_ reduction remains a major challenge.

Since 1985, the electrochemical reduction of CO_2_ has been well-known due to the first work by Professor Hori, in which various metal electrodes were used to reduce CO_2_ into gaseous and soluble products, such as CO, CH_4_, and HCOOH [2]. After this, much work has been undertaken to explore the effects of electrode materials, electrolyte concentration, reaction temperature, and PH, etc., on the reaction efficiency and possible products of different metal electrolytes that have been studied in-depth. In this regard, the crystal facet engineering of electrocatalysts has attracted great attention as a promising strategy for enhancing their catalytic performance and selectivity towards CO_2_ reduction. Crystal facet engineering refers to the control of the exposed crystal facets of catalysts, which can affect their electronic structure, surface chemistry, and adsorption properties. By tuning the crystal facets of electrocatalysts, their activity and selectivity towards CO_2_ reduction can be improved significantly [3]. Previous results have shown that the surface structure of a single crystal Cu directly determines the selectivity of the products that are generated by the CO_2_ reduction [4]. For example, ethylene has the highest selectivity as the main product on the Cu (100) surface, while methane is the main hydrocarbon product on the Cu (111) surface [5]. Raffaella Buonsanti et al. investigated the catalytic selectivity of the crystalline facets of Cu for the electrochemical reduction of CO_2_, and showed that the different crystalline facets of the catalyst had different catalytic reaction selectivities and suppressed the HER side reactions. The exposed (100) crystalline faces of Cu nanocubes were more selective for ethylene than Cu nanospheres, the exposed crystalline faces of Cu octahedra were (111), and methane was the main product of the CO_2_ conversion [6]. Wang et al. used a Cu nanocube as a precursor and etched Cu diamond dodecahedron nanocrystals with Se to expose the surface of the Cu (110) [7]. By controlling the etching time, etchant concentration, and etching temperature, the proportion of the (110) crystal surface on the surface of the Cu nanocrystals can be precisely controlled, thus the catalytic activity can be precisely controlled. The etched copper diamond dodecahedron exhibits a higher activity and selectivity in the electrochemical reduction of CO_2_ than the initial copper nanocube. Other metal catalysts also show a significant dependence on crystal facets in terms of their CO_2_ reduction selectivity [8,9,10]. For example, of the three low index crystalline surfaces of Ag (i.e., (100), (110), and (111)), Ag (110) has a rough and active site-rich surface, which provides the highest catalytic activity in the CO_2_ reduction reaction; therefore, Ag (110) has the highest CO selectivity [8].

In experiments, the popular ways of breaking the linear scaling include alloy and surface treatments [1]. Copper-based alloys have been reported to improve selectivity and efficiency [11,12,13]. For example, Feng et al. prepared a CuZn alloy to catalyze CO_2_ to ethylene with a Faradaic efficiency as high as 33.3% [14]. Other alloys without copper, such as Ag-Co [15], Ag-In [16], and Pd-Au [17], have also been reported for a high electivity over certain products, or for reducing CO_2_ to certain products that have never been observed on copper. Koper et al. found that a PdAu alloy was a good catalyst for the reduction of CO_2_ to long-chain hydrocarbons other than Cu, but no detailed catalytic mechanism was proposed [17]. DFT calculations can provide insights into the electronic structure, surface chemistry, and adsorption properties of electrocatalysts at the atomic level [18,19]. By combining DFT calculations with experimental measurements, the mechanism and kinetics of an electrocatalytic CO_2_ reduction can be elucidated in detail [20]. For CO_2_RR, Peterson and Nørskov tried to use activity descriptors to better understand copper’s unique properties for CO_2_RR and discovered new electrocatalysts with a higher performance [21]. Alexis T. Bell clarified the facet-dependent activity of Ag for the electrochemical reduction of CO_2_ to CO [22]. DFT calculations show that the reduction of CO_2_ to CO strongly depends on the structure of the crystal surface and the step surface being more active than the high-coordinated terraces. In addition, A. K. Srivastava have also shown that FLi_2_ superalkali is capable of reducing CO_2_ to a CO_2_^2-^ anion, as well by ab initio calculations [23]. Similarly, M. Czapla and P. Skurski demonstrated that the Sb_3_F_16_ superhalogen molecule is capable of ionizing CO_2_ systems and forming a stable ionic product [24]. Theoretical calculations provide more useful information for experiments.

The crystal facet engineering of electrocatalysts has shown a great potential for enhancing their catalytic performance and selectivity towards CO_2_ reduction. However, the mechanism and kinetics of electrocatalytic CO_2_ reduction on the different crystal facets of catalysts are still not fully understood. Therefore, further research is necessary to elucidate the relationship between the electrocatalytic CO_2_ reduction and the crystal facet engineering of catalysts, and to develop efficient and selective electrocatalysts for this CO_2_ reduction. In this paper, the possible pathways of CO_2_ to methane, methanol, and formic acid on a Pd_3_Au (111), (110), (100), and (211) facet was explored. It is hoped that the thermodynamic mapping of the C1 products on this alloy catalyst will create and provide guidance on the subsequent thermodynamic pathway mechanisms of the C2 and C3 long-chain products. The significance of this research lies in its potential to provide a sustainable and renewable approach to addressing the global energy and environmental challenges, and to promote the transition to a low-carbon economy.

## 2. Results and Discussions

**Figure 1:**
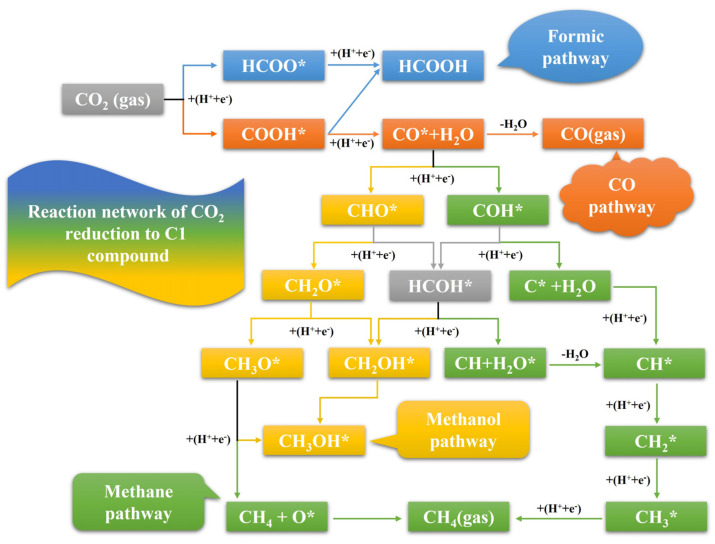
The overview of all possible pathways for CO_2_ reduction to C1 product.

The reaction network of the CO_2_ reduction to the C1 compounds (CO, HCOOH, CH_4_, and CH_3_OH) on the different Pd_3_Au surfaces that were included in this study are illustrated in Figure 1. All the reactions are carried out by the process of an electron−proton addition mechanism and start from the adsorption of CO_2_ gas on the surface. Subsequently, CO_2_* generates different products through a proton coupled electron transfer (PCET) process, which can produce a variety of multi-carbon products, from C1 to C5 [17].

Both the HCOOH and CO pathways involve two proton−electron pairs that are transferred. Through the HCOO* route, the CO_2_ is reduced to formate or formic acid as the product. In contrast, CO* is produced by the dehydration of the protonated COOH* intermediate. If the interaction between the CO* intermediate and the catalyst surface is weak, CO gas is the main product. Conversely, the strong binding strength between the CO* intermediate and the surface allows the CO* to continue its protonation to form COH* and CHO* intermediates, resulting in a variety of products [25]. On the different Pd_3_Au surfaces, methanol and methane products can be produced from both the COH* and CHO* pathways. The protonation of a CHO* intermediate on the C atom leads to a formaldehyde intermediate (CH_2_O*), or the protonation on the O atom leads to a HCOH* intermediate, both of which eventually lead to methane and methanol. Meanwhile, the protonation of COH* on the C atom produces a HCOH* intermediate and can also generate methane and methanol products, while the protonation of a COH* intermediate to a C* intermediate on the O atom can only generate methane [26]. In this work, all the possible reaction pathways of the electrocatalytic reduction of CO_2_ to C1 products were investigated.

### 2.1. The Properties of Different Pd_3_Au Surface

**Figure 2:**
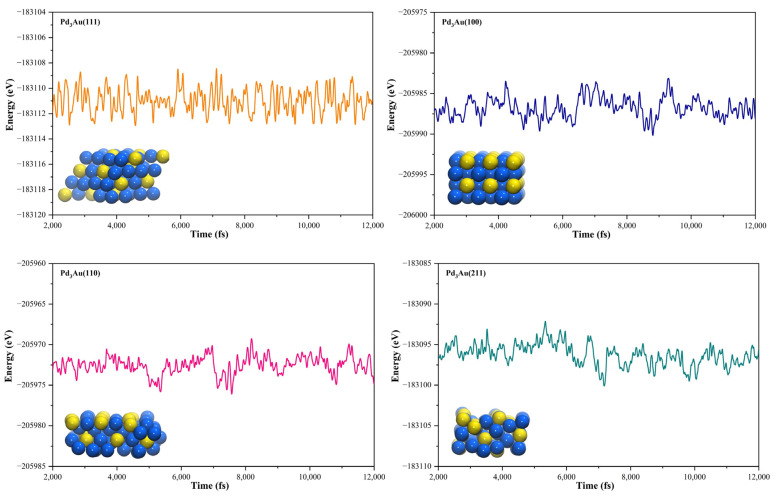
The total energy fluctuations of Pd_3_Au (111), (211), (100), and (110) surfaces, with progress of time (fs) in AIMD simulations at 1000 K. The snapshots of different Pd_3_Au surface geometric structures are displayed (Pd atom is represented by blue sphere and Au atom by yellow sphere).

The geometric structures of the Pd_3_Au (111), (211), (100), and (110) surfaces are shown in Figure S1 in Supplementary Material. It is well known that the low-index (111) and (100) surfaces are usually preferentially exposed in materials, due to their stability. In addition, due to the presence of steps, kinks, and edges structures, the (211) and (110) surfaces have a high reactivity [27,28,29]. To evaluate the stability of the Pd_3_Au (111), (211), (100), and (110) surfaces, the surface energy and surface formation energy of the different planes were calculated. As shown in Table S2, the results of the surface energy and surface formation energy are consistent, with Pd_3_Au (111) having the lowest surface energy and surface formation energy. This is also in agreement with the experimental results [30,31] for the most exposed (111) crystal faces in the Pd-Au alloy. In addition, the thermal stability of the Pd_3_Au (111), (211), (100), and (110) surfaces at T = 1000K was investigated by using an AIMD simulation. As shown in Figure 2, with an increase in the simulation time, the total energy of the four surfaces fluctuates within a certain range, while the structure does not change significantly, indicating that they also have an excellent stability beyond room temperature. Table S1 shows the adsorption energies of all the possible adsorption configurations that were examined for the different surfaces’ reaction intermediates in CO_2_RR. According to our previous studies [32], for each adsorbent, different adsorption sites (top, bridging sites, and face-centered cubes, etc.) were considered and the adsorbents were more likely to adsorb at the Pd-rich sites. Notably, the intermediates were rarely adsorbed at the Au-related sites, suggesting that the catalytically active sites were near the Pd atoms.

**Figure 3:**
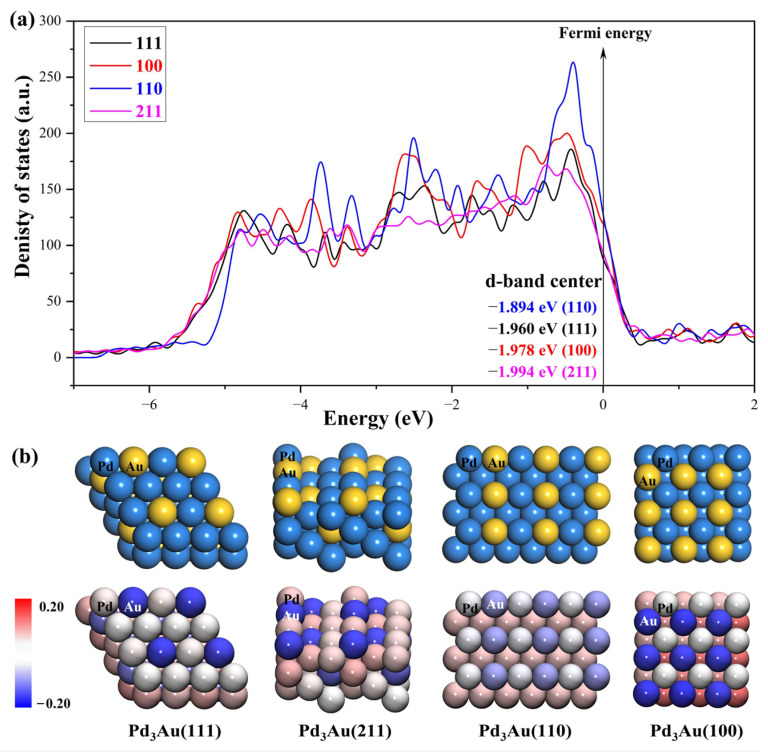
(**a**) d-orbital density of states of the Pd_3_Au (111), (100), (110), and (211) surfaces. (**b**) The optimized structures of different Pd_3_Au surfaces. Slab geometries with each atom are colored by the magnitude of their Bader charges.

The density of the states of the four surface structures are analyzed and the d-orbital population is observed. The density of the d-orbital states on the different surfaces is shown in Figure 3a. With the orientation moving from the (110) to the (211) surface, the distribution of the d-band orbital states shifts to the high energy region. Meanwhile, the change in the d-band center value has the same trend. The (211) surface has highest d-band center −1.984 eV, and the value of the d-band center varies by approximately 0.1 eV between the different surfaces. Furthermore, in order to determine the active sites on the different surfaces of the Pd_3_Au, the surface net charge (the number of valence electrons that are subtracted from the Bader charge of each atom, which are listed in Table S3) is determined by calculating the charge density at each site on the surface by a Bader charge analysis. As shown in Figure 3b, the slab geometries of each atom are colored by the magnitude of their Bader charges. For Pd_3_Au systems, the Au atoms at the top layer of the different surfaces are negatively charged and higher than the Au atoms at other locations. The Pd atoms at the top layer of the (111), (110), and (100) surfaces are close to neutral, while the Pd atoms at the (211) surface steps are positively charged. As we know, the charge distribution and morphology on the surface of the catalyst greatly affect the catalytic activity and selectivity, so the catalytic performance of the CO_2_ reduction on the different surfaces of Pd_3_Au will also vary.

### 2.2. Competitive HER Reaction and Formic Acid Formation

It is noteworthy that the hydrogen evolution reaction (HER) of the adsorbed protons is the most important competing reaction for the CO_2_ protonation and that the reaction rate is affected by the surface activity [33]. As can be seen from Table S4, the adsorption free energy of the H* species (ΔG_H*_) in the different surfaces of Pd_3_Au is negative, indicating that the HERs on the different surfaces of Pd_3_Au are controlled by the hydrogen desorption step. Furthermore, the lower the free energy of the adsorption of the H* species, the higher the water dissociation activity on the electrocatalyst [34]. Thus, the results suggest that the Pd_3_Au (111) and (211) surfaces can effectively promote H* production and preferentially enhance CO_2_RR, thereby inhibiting the HER. Then, the free energy changes in the HER and CO_2_ protonation were compared to determine the selectivity for the different surfaces. Tables S4 and S5 further reveal that the Pd_3_Au (100) surfaces have a good selectivity for the HER, while the CO_2_ protonation is dominant on the other surfaces. Surprisingly, by comparing the net surface charge and G on the different surfaces, it is found that increasing the net surface charge decreases the ΔG_H*_ and increases the selectivity for the hydrogen evolution reactions.

**Figure 4:**
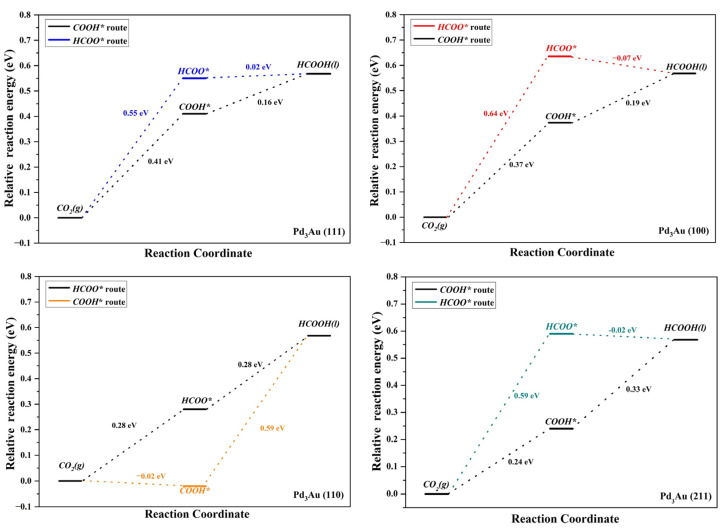
Free energy profiles for CO_2_RR to HCOOH on different Pd_3_Au surfaces (at 0 V vs. RHE).

Figure 1 illustrates the reaction pathway for the formation of HCOOH, in which the absorbed CO_2_ is reduced to HCOOH through two pathways [35,36,37]. One of these pathways is the formation of monodentate formate (COOH* route) intermediates via a proton transfer to the O atom of CO_2_, and the generation of HCOOH by dissociating hydrogen. The second method is to attach protons to the carbon atoms of CO to form bicarbonate (the HCOO* route), and then hydrogenate to form HCOOH. Although CO_2_ can produce HCOOH through both COOH* and CHOO* intermediates, the COOH* pathway competes with the formation of CO, while the CHOO* pathway is more selective and produces only HCOOH. The free energies of the CO_2_ gas reduction to HCOOH through these two pathways on the different Pd_3_Au surfaces are listed in Table S5 and Figure 4. The results show that COOH* intermediates on the Pd_3_Au surfaces are the main priority options for CO_2_ hydrogenation. Comparing the free energy of the continuous hydrogenation of the COOH intermediates, it is clear that COOH intermediates are more likely to be hydrogenated to generate CO rather than HCOOH on all the surfaces. At higher application potentials, the COOH* and HCOO* pathways are activated, and it can be observed that the CO_2_ on the Pd_3_Au (100) and (110) surfaces is reduced to HCOOH via the HCOO* intermediate, while the CO_2_ on the Pd_3_Au (111) and (211) surfaces is reduced to HCOOH via the COOH* pathway. Among them, the free energy barrier of the CO_2_ reduction to formic acid on the (110) surface is the lowest, and so the Pd_3_Au (110) surface has the best formic acid activity.

### 2.3. Formation and Competition of Methane and Formic Acid

Firstly, we investigated the cases of CO adsorption and protonation, where the reduction of CO_2_ to CO is a common start to the generation of methane and methanol productions, as shown in Figure 1. The appropriate CO adsorption strength determines the selectivity of the product [38]. If the CO adsorption energy is lower than 0.88 eV, the CO production is more favorable. If the adsorption is too strong, it will cause the CO poisoning of the catalyst and further affect the reaction. From the adsorption energy data in Table 1, it can be seen that the adsorption of CO on the four surfaces of Pd_3_Au is chemisorption, and that the interaction with the surface is moderate, which is conducive to the subsequent protonation translation. In addition, the interaction between CO* and the surfaces of Pd_3_Au obviously increases with the increase in the charge transfer. As depicted in Figure S3, the low energy barrier that is observed during the CO reduction process suggests that all four Pd_3_Au surfaces are capable of producing CO, without being hindered by COOH* poisoning. As mentioned above, the HER is the dominant reaction on the Pd_3_Au (100) surface. Consequently, this section focuses solely on the potential CORR occurring on the Pd_3_Au (111), (110), and (211) surfaces. The path from CO_2_ to the C1 product shows that CORR can be divided into two situations, as shown in Figure 1. In the first case, hydrogen atoms are added to the O atom of CO to form a COH* intermediate. Another case is where hydrogen atoms bond with C atoms to form CHO * intermediates. The further reduction of the two intermediates, CHO * and COH *, can lead to three pathways. The C * intermediate belongs only to the COH * pathway, which produces methane, while the CH_2_O * intermediate belongs only to the CHO * pathway, which produces methanol. By reducing to produce HCOH* intermediates, both the CHO * and COH * pathways can produce methane and methanol. All the possible reaction paths for the formation of methane and methanol on the four surfaces of Pd_3_Au were calculated, as shown in Figures S3 and S4.

Previous studies have reported that the protonation of adsorbed CO to generate adsorbed CHO*/CHO* is the PLS in the reduction of methane and methanol from CO_2_ on the different catalysts’ surfaces [25,28,39]. Interestingly, we found that the Pd_3_Au (211) surface with step structure breaks this relationship. On the Pd_3_Au (211) surface, both the COH* and CHO* pathways can produce methane, and the potential-limiting step (PLS) is CH* + H^+^ + e → CH_2_*, with a free energy barrier of 1.05 eV. However, methanol can only be generated through the COH* path, where the PLS is COH* + H^+^ + e → HCOH*, with a free energy barrier of 0.78 eV. Comparing the free energy barriers, it can be seen that the Pd_3_Au (211) surface has a good selectivity for methanol products.

According to Figure S3, the reduction of CO is most likely to occur via the COH* intermediates on the Pd_3_Au (211) and (111) surfaces, with reaction free energies of 0.57 and 0.52 eV, respectively. For the reduction of CO* to CHO * intermediates, their free energies are 1.11 and 1.01 eV, respectively, which are more difficult to overcome in thermodynamics. Interestingly, on the Pd_3_Au (110) surface, the free energies of the CO reduction to COH * and CHO * are 0.56 and 0.57 eV, respectively, which means that the selectivity of the product on the Pd_3_Au (110) surface is weak. The results show that, on the Pd_3_Au (110) surface, CO_2_ can be reduced to produce methane and methanol products via both the COH* and CHO* pathways, and the PLS is the same, both of which are CO* + H^+^ + e → COH*/CHO*. The selectivity should arise from the energy differences in the next intermediates, i.e., HCOH* and C-HOH*, as described by Kozuch and Shaik [39]. The reduction of COH* to form C-HOH* intermediates is evidently favored, leading to a facile CO_2_ reduction to methane on the Pd_3_Au (110) surface. At the same time, a similar situation occurs on the Pd_3_Au (111) surface. The results in Figure S3 show that the COH* pathway is the dominant pathway for the CO_2_ reduction on the (111) surface, and that the PLS for the formation of methane and methanol is CO* + H_+_ + e → COH*. Additionally, the free energies that are required for the protonation of COH* to produce HCOH * and C-H_2_O* intermediates are 0.52 and 0.50 eV, respectively. However, the free energy of the CH* intermediate that is produced by the HCOH* and C-H_2_O* intermediates is negative, which makes it easier to reduce to methane instead of methanol. Thus, the selectivity of the electrocatalytic reduction of CO_2_ on the Pd_3_Au (111) and (110) surfaces make it easier to produce methane.

**Figure 5:**
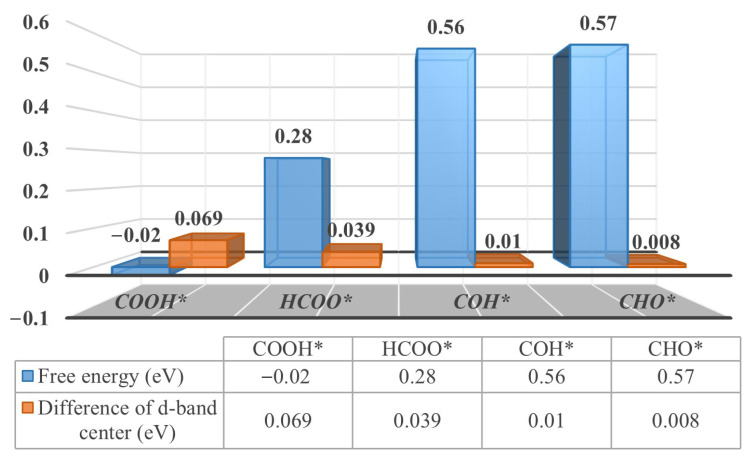
The formation free energy (eV) and d-band center difference (absolute value, eV) for adsorption of specific intermediates on Pd_3_Au (110) surfaces.

By calculating the d-band center difference of the top layer of the catalyst, where the adsorbent is located, it can be interpreted as the presence of free energy approaching reactions on the Pd_3_Au (111) and (110) surfaces. The relevant values of the d-band center are summarized in Table S5. If the reaction steps involve a transformation of the adsorbed species, the selectivity of the reaction is determined by the differences between the d-band centers at the top of the catalyst, where the reactant and product are located. The greater the difference in the top-layer d-band centers between the product and the reactant, the lower the free energy of the formation, resulting in a greater selectivity of the product. For example, the protonation of CO_2_ produces HCOO* and COOH* intermediates on the Pd_3_Au (110) surface, as shown in Figure 5. The absolute differences between the top d-band centers of the products (COOH* and HCOO* intermediates) and the reactants (CO_2_* intermediates) are 0.069 and 0.039 eV, respectively. Meanwhile, the formation free energies of the COOH* and HCOO* intermediates are −0.02 and 0.28 eV, respectively. Compared to the HCOO* intermediates, the protonating of CO_2_ makes it easier to use to produce COOH* intermediates. For the protonation of CO*, the absolute values of the difference between the top d-band centers of the COH and CHO* intermediates and the CO* intermediate are very close, at 0.01 and 0.008 eV, respectively. Accordingly, the formation free energies of the COH* and COH* intermediates are very close to each other. This correlation is also applicable to the Pd_3_Au (111) and (211) surfaces, as illustrated in Figures S6 and S7.

## 3. Computational Method

### 3.1. Models

The Pd-Au alloy was constructed by replacing one Pd atom in the corner of an fcc Pd cell with an Au atom, in which the atom ratio of the alloy was Pd: Au = 3: 1. The lattice constant of the alloy was 4.028 Å after the optimization of the bulk alloy. Based on the optimized bulk structure, the (111), (110), (100), and (211) surfaces of the Pd_3_Au structure were then modeled by a four-layer slab, using a (2 × 2) supercell with the top two layers relaxed and the rest fixed to the bulk distances. To avoid the possible interaction between the layers, a 15.0 Å vacuum region along the c-axis of a slab model was introduced. The Brillouin zone for the geometry optimization of the (111), (110), (100), and (211) surfaces of the Pd_3_Au was sampled with a Gamma-centered k-mesh of 3 × 3 × 1, 3 × 2 × 1, 3 × 3 × 1, and 3 × 2 × 1, respectively. For calculating the density of the states, the Gamma-centered k-mesh was increased to 5 × 5 × 1. To evaluate the stability of the different planes, the ab initio molecular dynamics (AIMD) simulations were performed using a canonical ensemble (NVT), with the Nosé thermostat at 1000 K with a 1 fs time step and an overall time scale of 12 ps. The AIMD simulation, using DS-PAW software, was integrated in the Device Studio program [40].

### 3.2. DFT Calculations

All the calculations were performed within the framework of the density functional theory (DFT), as implemented by the Vienna Ab initio Simulation Package (VASP) [41,42]. The energy cutoff for the plane wave expansion was set to 400 eV. The convergence criterion for the self-consistent iteration was set to 1 × 10^−5^ eV atom^−1^, and the systems were fully relaxed until the final force on each atom was less than 0.03 eV Å^−1^. A projector-augmented wave (PAW) was used to account for the core-valence interactions [43,44] within the exchange–correlation function and was described by Perdew, Burke, and Ernzerhof (PBE) [45]. The D3 correction of Grimme [46,47] was adopted to compensate for the lack of a van der Waals interaction description in the GGA functional. The Brillouin zone for the energies calculation of the (111), (110), (100), and (211) surfaces was sampled with a Gamma-centered k-mesh of 3 × 3 × 1, 3 × 2 × 1, 3 × 3 × 1, and 3 × 2 × 1, respectively. In addition, the implicit solvent model was used for all the free energies calculations to consider the influence of the solvation effect, which was realized by the VASPsol program [48,49]. The dielectric constant was set to 78.4 to simulate the effects of the electrostatics, cavitation, and dispersion on the interaction of the metal surface and water. The Bader charge analysis was used [50,51] to observe the behavior of the electronic charge transfer in the atoms on the outer surface. The change in the Gibbs free energy of the reactions (Δ*G*) that were involved in this work was calculated using the computational hydrogen electrode (CHE) model [52]. The free energy, depending on the applied potential Δ*G*(U), is calculated by the equation:

Δ*G*(U) = Δ*G*(0V) − *neU*

where *e* and are the number of electrons and transferred electrons, respectively. Δ*G*(0V) is the Gibbs free energy change of each reaction step without the applied potential. The limit potential (*U*_L_) is obtained from the equation:

*U*_L_ = −Δ*G*_PLS_/*e*

where Δ*G*_PLS_ is the free energy change for the potential-limiting step (PLS) along each CO_2_RR pathway. In this study, we only consider the thermodynamics electrocatalysis, and based on the Brønsted–Evans–Polanyi (BEP) relationship, it is assumed that the PLS corresponds to the kinetic rate limiting step [53,54]. The details concerning the slab model, adsorption energies, and free energy corrections are provided in the supporting information.

## 4. Conclusions

In this work, the mechanism of the electrochemical reduction of CO_2_ on four different surfaces of Pd_3_Au alloy bimetal catalysts was studied by using the density functional theory. The results showed that the different d-band populations, charge distributions, and morphologies on the four surfaces of the Pd_3_Au bimetal alloy catalysts resulted in different catalytic activities and selectivities on the different surfaces of the catalysts. A charge analysis showed that the increase in the net charge on the surface of the alloy decreased the adsorption of the H atom on the catalyst, thereby increasing the selectivity for the HER. The Pd_3_Au (100) surface, with the largest net surface charge, had a good selectivity for the HER. At higher application potentials, the Pd_3_Au (110) surface had the best formic acid activity. Among the four surfaces of the Pd_3_Au bimetal catalyst, the (211) surface had a good selectivity for methanol production from CO_2_RR. The electron structure analysis further showed that the difference between the d-band center at the top of the catalyst and the product determined the selectivity of the adsorbate conversion reaction. These findings will be useful in the design and development of more efficient and selective electrocatalytic CO_2_ reduction catalysts.

## Figures and Tables

**Table 1:** CO adsorption energy, charge transfer, and charge density differences on different Pd_3_Au surface.

	E_ad_(CO) [eV]	Charge Transfer [|e|]	Charge Density Difference
Pd_3_Au (111)	−2.26	0.21	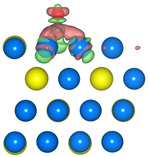
Pd_3_Au (100)	−1.68	0.11	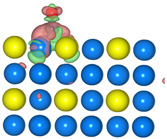
Pd_3_Au (110)	−1.96	0.17	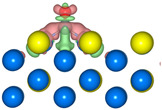
Pd_3_Au (211)	−2.27	0.22	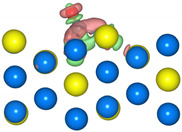

## Data Availability

Not applicable.

## Supplementary Materials

The following supporting information can be downloaded at: https://www.mdpi.com/article/10.3390/molecules28073169/s1, Table S1: The calculated adsorption energies (eV) of key adsorbates on the Pd3Au (111), (100), (110) and (211) crystal facets. Table S2: The surface energy of different Pd3Au surface. Table S3: The atom’s net charge calculated by the Bader charge analysis. (Net charge = Valence charge − Total Bader charge, the valence charges of Pd and Au are 10 and 11, respectively.). Table S4: The adsorption free energy (eV) of H atoms adsorbed on on different Pd3Au surface. Table S5: The free energy (eV) of CO2 reduction to HCOOH and CO on different Pd3Au surface. Table S6 The top-layer d-band center and d-band center difference (absolute value) for adsorption of specific intermediates on different Pd3Au surfaces. Figure S1: The optimized structures of different Pd3Au facets (the blue ball represents Pd atom and yellow ball represents Au atom). Figure S2: Free-energy profiles for CO2RR to COH*/CHO* on different Pd3Au surface (at 0 V vs. RHE). Figure S3: Free-energy profiles for CO2RR to CH4 on different Pd3Au surface (at 0 V vs. RHE). Figure S4: Free-energy profiles for CO2RR to CH3OH on different Pd3Au surface (at 0 V vs. RHE). Figure S5: The formation free energy and d-band center difference (absolute value) for adsorption of specific intermediates on Pd3Au (111) surfaces. Figure S6: The formation free energy and d-band center difference (absolute value) for adsorption of specific intermediates on Pd3Au (211) surfaces.
