# Supplementary material for: The Facet Dependence of CO2 Electroreduction Selectivity on a Pd3Au Bimetallic Catalyst: A DFT Study
Source: Molecules. 2023 Apr 2;28(7):3169. doi: 10.3390/molecules28073169 (PMC10095644; doi:10.3390/molecules28073169)
Supplement: Supplementary file 1 [file molecules-28-03169-s001.zip › molecules-2332412-supplementary.pdf]

Facet Dependence of CO_2 Electroreduction Selectivity on Pd_3Au Bimetallic Catalyst: A DFT Study

Ming Zheng [1], Xin Zhou [1]*, Yixin Wang [1], Gang Chen [1], Mingxia Li [2]*
[1] MIIT Key Laboratory of Critical Materials Technology for New Energy Conversion and Storage,
School of Chemistry and Chemical Esngineering, Harbin Institute of Technology, Harbin150001
P. R. China
[2] School of Chemistry and Materials Science, Key Laboratory of Functional Inorganic Material
Chemistry, Ministry of Education of the People's Republic of China, Heilongjiang University,
Harbin 150080, P. R. China
*Corresponding authors: zhoux@hit.edu.cn and limingxia@hlju.edu.cn

Computational methods and model

Free energy corrections

In order to investigate the thermodynamic reaction path, the free energy profiles of the hydrogen evolution reaction (HER) and CO_2RR were computed using the computational hydrogen electrode (CHE) model proposed by Nørskov and co-workers[1]. All free energies (ΔG) were calculated by the following equation:

$$\Delta G = \Delta E_{DFT} + \Delta ZPE - T\Delta S$$

where ΔE_{DFT} is the energy difference between reactants and products, obtained from DFT calculations; ΔZPE and ΔS are the energy differences in zero-point energy and entropy; T is 298.15 K. ZPE was calculated from vibrational modes obtained within the harmonic oscillator approximation. Vibrational contributions to the entropies for substrate were considered to be inconsequential and counted as zero in the part of TΔS.

For molecules, the vibrational contributions to the entropies were calculated from standard thermodynamic tables at 298.15 K and 1 atm.

Adsorption energy

Various intermediates adsorbed on different sites on the surface were considered. Generally, the adsorption sites can be classified into three types: top, bridge, and hollow sites. Hollow site includes fcc-like and hcp-like sites in close-packed crystal structure.

Configurations of all possible intermediates were obtained by performing a series of geometry optimization on various adsorption sites and the adsorption energies of them were calculated. Adsorption energy is calculated by the following equation:

$$E_{ads} = E_{adsorbate\text{-}slab} - E_{adsorbate} - E_{slab}$$

where $E_{adsorbate\text{-}slab}$, E_{slab} and $E_{adsorbate}$ are the total energies of the adsorbed, bare slabs and gaseous molecules, respectively.

Slab model

In theoretical research, the Au-Pd system compounds were found at the experimentally known compositions. The UPb[2, 3] prototype structure (Nr40[4]) is predicted the most stable phase for AuPd. But experimental proof for the existence of this structure is still lacking, probably due to the low order-disorder temperature. The phase diagram of the Au-Pd system has ordered $L1_2$ structures ($L1_2$ is index by Strukturbericht designation and the prototype is $AuCu_3$ structure) for Au_3Pd and $AuPd_3$ compositions, which already identified by experiment.[5]

In the experiment, Marco Valenti et al[6] measured the faradaic efficiencies for H_2 of the five synthesized Pd-Au alloy electrodes at −0.5 V vs RHE. The faradaic efficiencies for H_2 in $Au_{25}Pd_{75}$ is the minimum, which indicating $Au_{25}Pd_{75}$ could effective suppresses H_2 evolution reaction (HER). In addition, with the increasing the Pd content, the partial current density of H_2 decreases, which also indicate $Au_{25}Pd_{75}$ could effectively suppress HER. It can thus be seen, when the Pd Au ratio was 1:3, the material could effectively suppress HER.

The surface segregation of Pd-Au system is a complex process, which is affected by many factors (such as temperature=e and adsorption molecule[7, 8]). Dragana D et al.[9] found that in the case of CO adsorption, the Au surface segregation of Pd_3Au (111) becomes endothermic, which indicates that the surface maintains a bulk composition in the presence of CO. In addition, it is reported that the surface enrichment of PdAu nano-clusters in the presence of CO is observed by Diffuse Reflectance Infrared Fourier Transform Spectroscopy (DRIFTS), and DFT calculations show that Pd atoms prefer to low-coordination at the edges of nano-clusters[7]. Gao et al. reported similar results using polarization modulated infrared reflectance absorption spectroscopy (PM-IRAS). When the CO pressure is higher than ~0.1 Torr, Pd segregation is greatly enhanced, forming continuous Pd sites[8]. However, some experimental reports have shown that surface atoms do not segregate in the presence of strongly adsorbed species during the CO_2RR. Marco Valenti et al[6] collected XPS spectra of PdAu alloys before and after electrolysis at −0.5 V vs RHE for 20 min. The results show that there are no significant changes in the shape of the valence band are noted, which suggests that no phase segregation occurred during the reaction that could affect the local electronic properties of the electrode surface. In addition, the CV spectrum shows that the reduction peaks of the alloy do not overlap with the pure components, indicating that there is no region containing pure Au or Pd on the surface of the alloy electrode. It can be seen that the segregation of alloys is very complex, especially in the electrochemical environment. In this wok, we mainly explore the mechanism of CO_2 reduction on specific electronic structure PdAu alloys, even though partial segregation of surface atoms in really experiment possible, the material still retains its specific electronic structure. Thus, the Pd-Au slab model without segregation was used in this work.

Table S1 The calculated adsorption energies (eV) of key adsorbates on the Pd_3Au (111), (100), (110) and (211) crystal facets.

	(111)	(100)	(110)	(211)
CO_2*	-0.28	-0.28	-0.19	-0.35
COOH*	-2.16	-2.16	-2.60	-2.37
HCOO*	-2.49	-2.37	-2.75	-2.44
HCOOH	-0.59	-0.27	-0.66	-0.37
CO*	-2.26	-1.68	-1.96	-2.27
COH*	-4.63	-3.67	-4.21	-4.60
CHO*	-2.47	-2.35	-2.73	-2.60
C*	-6.92	-7.20	-7.92	-7.08
CH*	-6.50	-6.11	-6.61	-6.54
CH2*	-3.99	-3.41	-3.99	-3.19
CH3*	-1.95	-1.85	-2.12	-2.00
CH4	-0.26	-0.27	-0.07	-0.28
HCOH*	-3.30	-3.03	-3.22	-2.95
OCH2*	-0.59	-0.45	-0.93	-0.63
OCH3*	-1.77	-1.81	1.38	-1.83
CH2OH*	-2.05	-1.95	-2.17	-1.85
CH3OH	-0.61	-0.59	-0.62	-0.51

Table S2 The surface energy of different Pd_3Au surface.

	(111)	(100)	(211)	(110)
Surface energy (J/m^2)	1.91	2.14	2.16	2.23
Surface formation energy (eV)	-0.23	-0.12	-0.03	0.12

Table S3 The atom's net charge calculated by the Bader charge analysis. (Net charge = Valence charge − Total Bader charge, the valence charges of Pd and Au are 10 and 11, respectively.)

	Top layer				2nd layer			
	(100)	(110)	(111)	(211)	(100)	(110)	(111)	(211)
Pd	-0.016	0.027	-0.023	-0.053	-0.093	-0.058	-0.069	-0.080
Au	0.164	0.085	0.160	0.156	—	—	0.175	0.123
Total	1.330	1.010	0.361	0.390	-1.679	-1.050	-0.395	-0.467

Table S4 The adsorption free energy (eV) of H atoms adsorbed on on different Pd_3Au surface.

	(111)	(100)	(211)	(110)
ΔG_{H*}	-0.48	-0.16	-0.37	-0.22

Table S5 The free energy (eV) of CO_2 reduction to HCOOH and CO on different Pd_3Au surface.

Reaction Steps	(111)	(100)	(211)	(110)
CO_2 + H^++ e → COOH*	0.41	0.64	0.24	-0.02
COOH* + H^++ e →HCOOH	0.16	-0.07	0.33	0.59
CO_2 + H^++ e →HCOO*	0.55	0.37	0.59	0.28
HCOO* + H^++ e →HCOOH	0.02	0.19	-0.02	0.28
COOH* + H^++ e→ CO-HOH*	-1.40	-0.69	-0.26	-0.72

Table S6 The top-layer d-band center and d-band center difference (absolute value) for adsorption of specific intermediates on different Pd_3Au surfaces.

		(110)	(111)	(211)
d-band center of top layer	CO_2*	-2.083	-2.005	-2.233
	COOH*	-2.152	-2.077	-2.266
	HCOO*	-2.122	-2.032	-2.249
	CO*	-2.144	-2.075	-2.27
	COH*	-2.134	-2.125	-2.321
	CHO*	-2.152	-2.075	-2.291
difference of d-band center (Absolute value)	COOH*	-0.069	-0.072	-0.033
	HCOO*	-0.039	-0.027	-0.016
	COH*	0.01	-0.05	-0.051
	CHO*	-0.008	0	-0.021

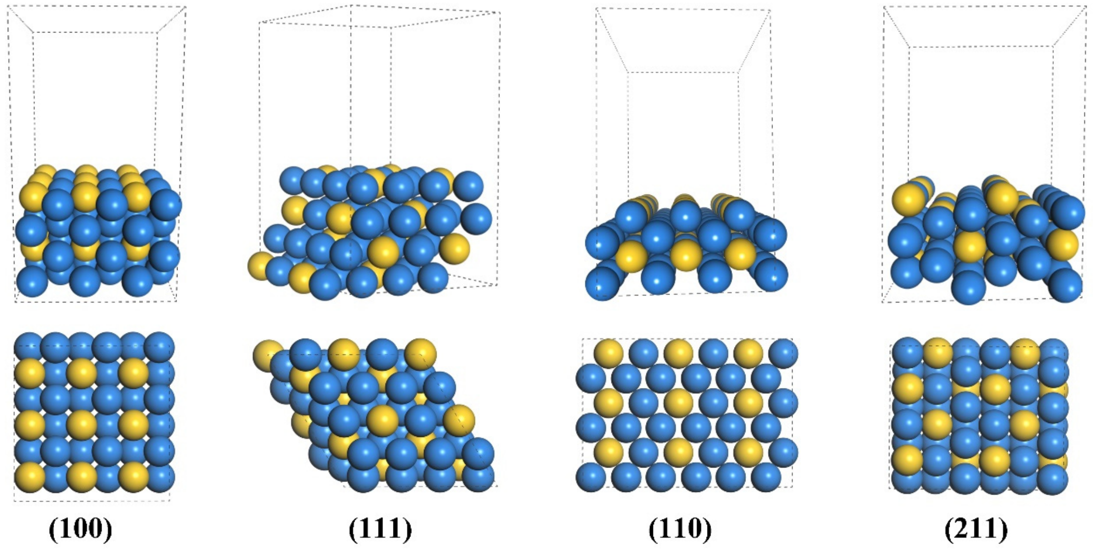

Figure S1 The optimized structures of different Pd_3Au facets (the blue ball represents Pd atom and yellow ball represents Au atom).

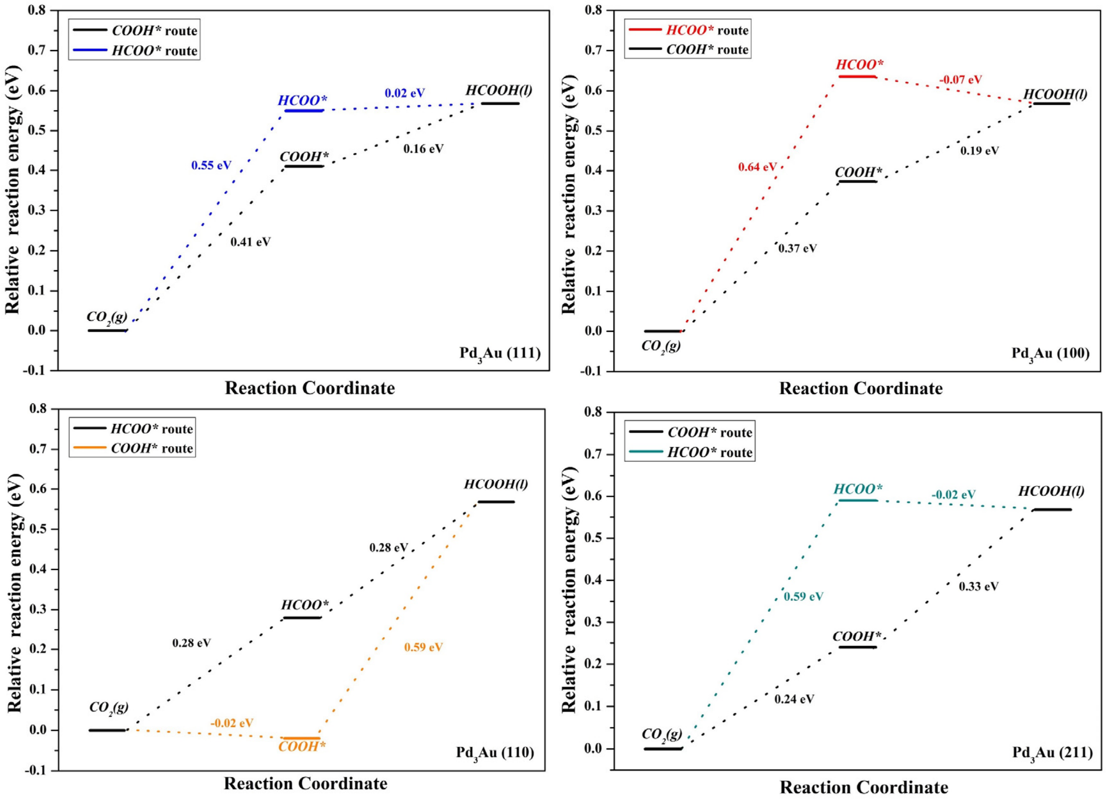

Figure S2 Free-energy profiles for CO_2RR to COH*/CHO* on different Pd_3Au surface (at 0 V vs. RHE).

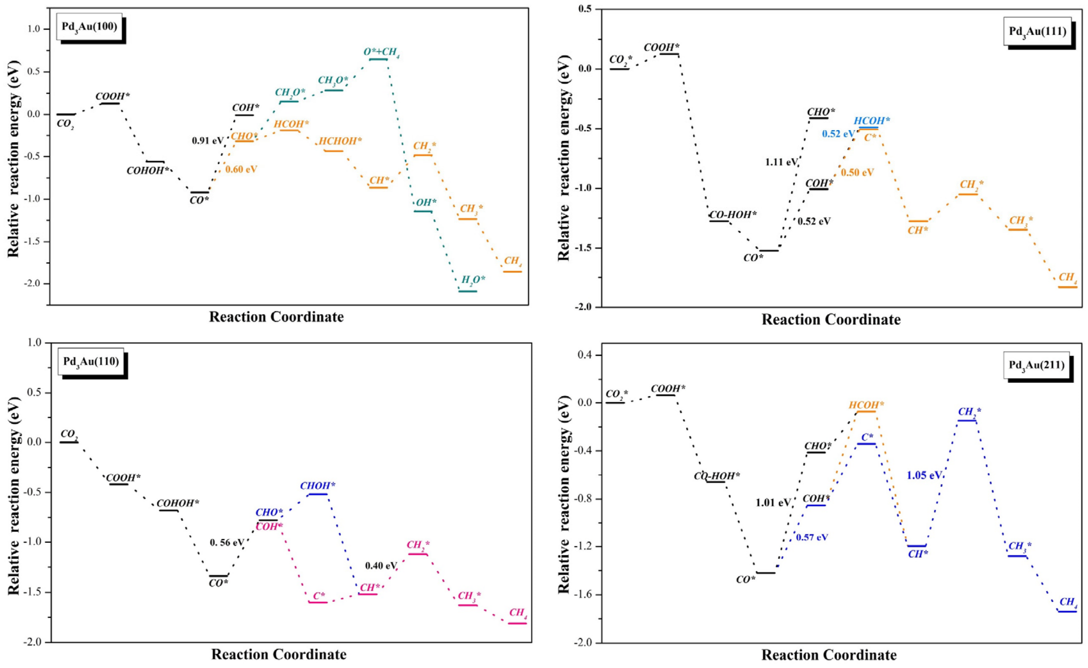

Figure S3 Free-energy profiles for CO_2RR to CH_4 on different Pd_3Au surface (at 0 V vs. RHE).

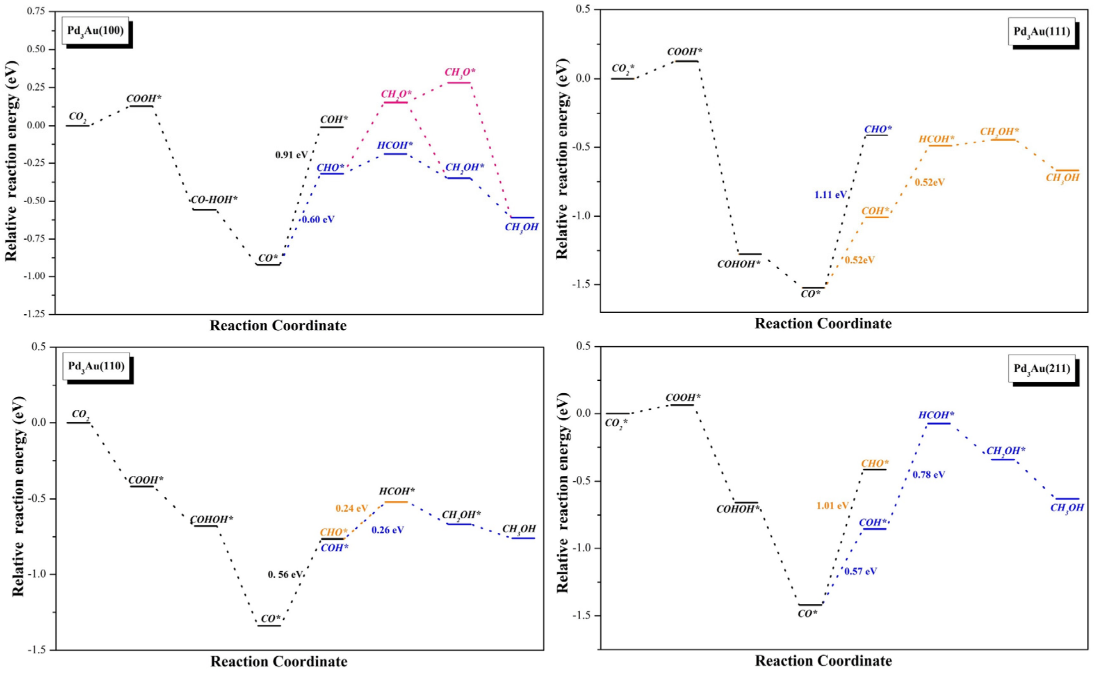

Figure S4 Free-energy profiles for CO_2RR to CH_3OH on different Pd_3Au surface (at 0 V vs. RHE).

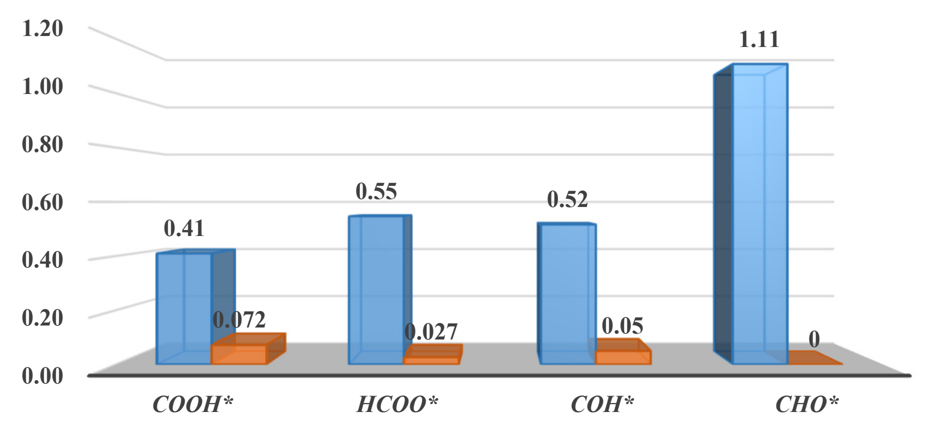

	COOH*	HCOO*	COH*	CHO*
Free energy (eV)	0.41	0.55	0.52	1.11
Difference of d-band center	0.072	0.027	0.05	0

Figure S5 The formation free energy and d-band center difference (absolute value) for adsorption of specific intermediates on Pd_3Au (111) surfaces.

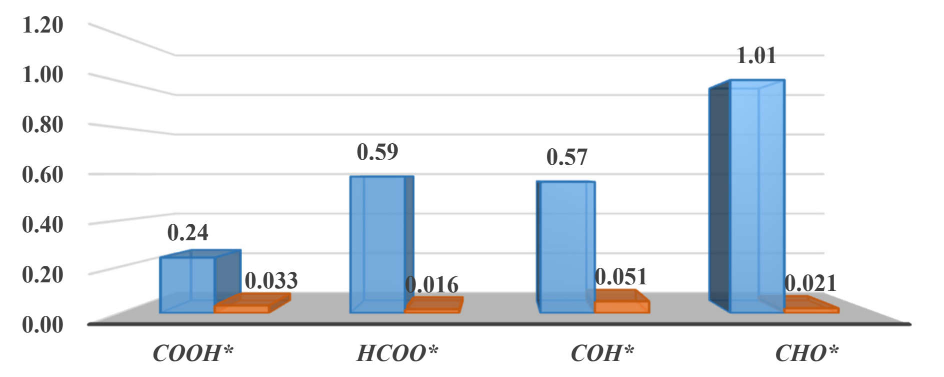

	COOH*	HCOO*	COH*	CHO*
Free energy (eV)	0.24	0.59	0.57	1.01
Difference of d-band center	0.033	0.016	0.051	0.021

Figure S6 The formation free energy and d-band center difference (absolute value) for adsorption of specific intermediates on Pd_3Au (211) surfaces.

References

[1] A.A. Peterson, F. Abild-Pedersen, F. Studt, J. Rossmeisl, J.K. Nørskov, How copper catalyzes the electroreduction of carbon dioxide into hydrocarbon fuels, Energy & Environmental Science, 3 (2010) 1311-1315.

[2] D.Y. Jo, H.C. Ham, K.-Y. Lee, Facet-dependent electrocatalysis in the HCOOH synthesis from CO_2 reduction on Cu catalyst: a density functional theory study, Applied Surface Science, 527 (2020) 146857.

[3] A. Brown, The crystal structures of ThPb3, UPb3, ThPb and UPb, Acta Crystallographica, 14 (1961) 856-860.

[4] J. Kanamori, Y. Kakehashi, Conditions for the existence of ordered structure in binary alloy systems, J. Phys. Colloques 38 (1977) C7-274-C7-279.

[5] M.H.F. Sluiter, C. Colinet, A. Pasturel, Ab initio calculation of the phase stability in Au-Pd and Ag-Pt alloys, Physical Review B, 73 (2006) 174204.

[6] M. Valenti, N.P. Prasad, R. Kas, D. Bohra, M. Ma, V. Balasubramanian, L. Chu, S. Gimenez, J. Bisquert, B. Dam, W.A. Smith, Suppressing H2 Evolution and Promoting Selective CO_2 Electroreduction to CO at Low Overpotentials by Alloying Au with Pd, ACS Catalysis, 9 (2019) 3527-3536.

[7] B. Zhu, G. Thrimurthulu, L. Delannoy, C. Louis, C. Mottet, J. Creuze, B. Legrand, H. Guesmi, Evidence of Pd segregation and stabilization at edges of AuPd nano-clusters in the presence of CO: A combined DFT and DRIFTS study, Journal of Catalysis, 308 (2013) 272-281.

[8] F. Gao, Y. Wang, D.W. Goodman, CO oxidation over AuPd(100) from Ultrahigh Vacuum to Near-Atmospheric Pressures: CO Adsorption-Induced Surface Segregation and Reaction Kinetics, The Journal of Physical Chemistry C, 113 (2009) 14993-15000.

[9] D.D. Vasić Anićijević, V.M. Nikolić, M.P. Marčeta Kaninski, I.A. Pašti, Structure, chemisorption properties and electrocatalysis by Pd_3Au overlayers on tungsten carbide – A DFT study, International Journal of Hydrogen Energy, 40 (2015) 6085-6096.
